# Teaching scalpel skills, does it make *sense*? A comparison of sensory and motor practice methods

**DOI:** 10.1186/1757-1146-8-S2-O6

**Published:** 2015-09-22

**Authors:** Ryan S Causby, Michelle N McDonnell, Lloyd Reed, Susan Hillier

**Affiliations:** 1 International Centre for Allied Health Evidence, University of South Australia, Adelaide, SA, 5000, Australia; 2 School of Clinical Sciences, Queensland University of Technology, Kelvin Grove, Qld, 4059, Australia

**Keywords:** Psychomotor skills, Motor skill learning, Dexterity, Teaching methods

## Background

Teaching psychomotor skills requiring high levels of dexterity can be difficult, particularly if students lack innate ability. Furthermore this can be a safety issue when real subjects are involved. Previous studies have found sensory awareness training can improve dexterity over the short-term. Therefore it seems prudent to determine if this strategy can provide an effective alternative to current teaching strategies.

## Methods

A randomised controlled trial with 2nd year students from UniSA and QUT (n=44) was used to compare sensory awareness training, motor practice training with a scalpel or standard teaching practice for 3 weeks. Outcomes included psychological measures (Intrinsic Motivation Inventory) and dexterity measures (Purdue pegboard, Grooved pegboard test, Grip-lift task).

## Results

A significant group difference was evident for perceived competence (self-efficacy)(p=0.019), lift duration (dominant hand)(p=0.004) and maximum grip force (dominant hand)(p=0.04) for the grip-lift task in favour of the motor practice group. No other significant group differences were found. Handedness, location and group by gender differences were evident on some of the test outcomes.

## Conclusions

Sensory awareness training does not appear to provide a more effective teaching strategy for increasing dexterity. Instead the provision of additional motor practice has a small benefit including facilitating an increase in the perceived competence (self-efficacy). This may involve simple motion replication and practice on inanimate objects, which may be beneficial and a safer option during the early stages of motor learning. Further research may be warranted utilising alternate methods of sensory awareness training, evaluating long term effects (retention) with greater participant numbers.

